# Spectrum-Malaria: a user-friendly projection tool for health impact assessment and strategic planning by malaria control programmes in sub-Saharan Africa

**DOI:** 10.1186/s12936-017-1705-3

**Published:** 2017-02-10

**Authors:** Matthew Hamilton, Guy Mahiane, Elric Werst, Rachel Sanders, Olivier Briët, Thomas Smith, Richard Cibulskis, Ewan Cameron, Samir Bhatt, Daniel J. Weiss, Peter W. Gething, Carel Pretorius, Eline L. Korenromp

**Affiliations:** 1 Avenir Health, Geneva, 1 route de Morillons/150 Route de Ferney (WCC, office 164), PO box 2100, 1211 Geneva 2, Switzerland; 2 Avenir Health, Glastonbury, USA; 3 Swiss Tropical and Public Health Institute, Basel, Switzerland; 4 University of Basel, Basel, Switzerland; 5 World Health Organization Global Malaria Programme, Geneva, Switzerland; 6 Oxford Big Data Institute, Li Ka Shing Centre for Health Information and Discovery, University of Oxford, Oxford, UK; 7 Department of Infectious Disease Epidemiology, Imperial College London, London, UK

**Keywords:** Malaria, Prevention, Treatment, Mortality, Morbidity, Health impact, Programmes, Policy evaluation, Strategic planning

## Abstract

**Background:**

Scale-up of malaria prevention and treatment needs to continue but national strategies and budget allocations are not always evidence-based. This article presents a new modelling tool projecting malaria infection, cases and deaths to support impact evaluation, target setting and strategic planning.

**Methods:**

Nested in the Spectrum suite of programme planning tools, the model includes historic estimates of case incidence and deaths in groups aged up to 4, 5–14, and 15+ years, and prevalence of *Plasmodium falciparum* infection (*Pf*PR) among children 2–9 years, for 43 sub-Saharan African countries and their 602 provinces, from the WHO and malaria atlas project. Impacts over 2016–2030 are projected for insecticide-treated nets (ITNs), indoor residual spraying (IRS), seasonal malaria chemoprevention (SMC), and effective management of uncomplicated cases (CMU) and severe cases (CMS), using statistical functions fitted to proportional burden reductions simulated in the *P. falciparum* dynamic transmission model OpenMalaria.

**Results:**

In projections for Nigeria, ITNs, IRS, CMU, and CMS scale-up reduced health burdens in all age groups, with largest proportional and especially absolute reductions in children up to 4 years old. Impacts increased from 8 to 10 years following scale-up, reflecting dynamic effects. For scale-up of each intervention to 80% effective coverage, CMU had the largest impacts across all health outcomes, followed by ITNs and IRS; CMS and SMC conferred additional small but rapid mortality impacts.

**Discussion:**

Spectrum-Malaria’s user-friendly interface and intuitive display of baseline data and scenario projections holds promise to facilitate capacity building and policy dialogue in malaria programme prioritization. The module’s linking to the OneHealth Tool for costing will support use of the software for strategic budget allocation. In settings with moderately low coverage levels, such as Nigeria, improving case management and achieving universal coverage with ITNs could achieve considerable burden reductions. Projections remain to be refined and validated with local expert input data and actual policy scenarios.

**Electronic supplementary material:**

The online version of this article (doi:10.1186/s12936-017-1705-3) contains supplementary material, which is available to authorized users.

## Background

Effective malaria prevention and treatment interventions have been scaled-up substantially with increasing national and donor funding since the early 2000s. Between 2000 and 2015, malaria incidence rates fell 37% globally, and malaria mortality rates by 60%, with even greater declines in Africa, the highest-burden region [1]. This was likely a combined result of improved malaria control and improving socio-economic factors [2, 3].

To sustain these improvements, the World Health Organization (WHO) global technical strategy for malaria recommends further scale-up to universal coverage with suitable preventive and curative interventions [4]. Funding for malaria has now plateau-ed however, placing more emphasis on prioritizing interventions with the most impact. While most countries focus on WHO-recommended proven effective interventions, national strategies and plans vary considerably in budget allocations across interventions, and rationales for mixes of interventions are often not explicit [5].

For many health areas, strategic decision-making is supported by simple, user-friendly programme planning tools that project impacts and costs of user-defined scale-up scenarios. One such tool is the Spectrum suite of policy models, used by over 120 low- and middle-income countries for estimating burdens, trends, service needs, and programme impact for family planning, HIV/AIDS, tuberculosis, and/or maternal and child health services [6–10]. As of 2015, Spectrum did not have a malaria module, although a simple linear, coverage-impact function included in the lives saved tool (LiST) projects impacts of selected malaria interventions on under-five mortality [11–13].

To extend and complement malaria projections available through LiST, a malaria impact module was developed in Spectrum, with the following refined features:

- Impact projections reflect population-dynamic effects over short- and long-term, including shifts in endemicity, acquired immunity and cohort effects resulting when interventions lower malaria transmission;
- Health outcomes include malaria mortality and case incidence, each in children aged up to 4 years old, 5–14 years and adults 15+ years, as well as the prevalence of *Plasmodium falciparum* infection (*Pf*PR) in children 2–9 years, as another common programme performance and impact indicator;
- The model can be applied at national as well as at province levels, accounting for heterogeneity among provinces within a country in baseline endemicity and intervention coverages, and their implications for expected health impact.

This paper describes the conceptual approach, data inputs and assumptions of Spectrum-Malaria, and illustrates the tool’s functioning with scenario projections hypothesized for Nigeria. Results are discussed considering future refined applications as are being initiated with national programme planners, and expectations for evidence-based programme planning articulated by national policy makers, international technical advisors and donors.

## Methods

### Conceptual design and modelling approach

Spectrum-Malaria was designed for application in 43 sub-Saharan African countries with stable transmission of *P. falciparum* as the predominant species. Health effects of intervention scale-up in Spectrum are based on statistical functions fitted to simulations done in the OpenMalaria dynamic transmission model in the version Schema 32 [14] calibrated for *P. falciparum* [15]. Simulations and multivariate regression functions included as endemicity-related impact determinants: the annual entomological inoculation rate (EIR), the *Pf*PR at 2000–2002, and the seasonality in malaria transmission. Simulated impacts for user-specified scale-up were statistically summarized for usage of insecticide-treated nets (ITNs), indoor residual spraying (IRS), seasonal malaria chemoprevention (SMC), and effective management of uncomplicated cases (CMU) and severe cases (CMS), as detailed in [16].

**Fig. 1:**
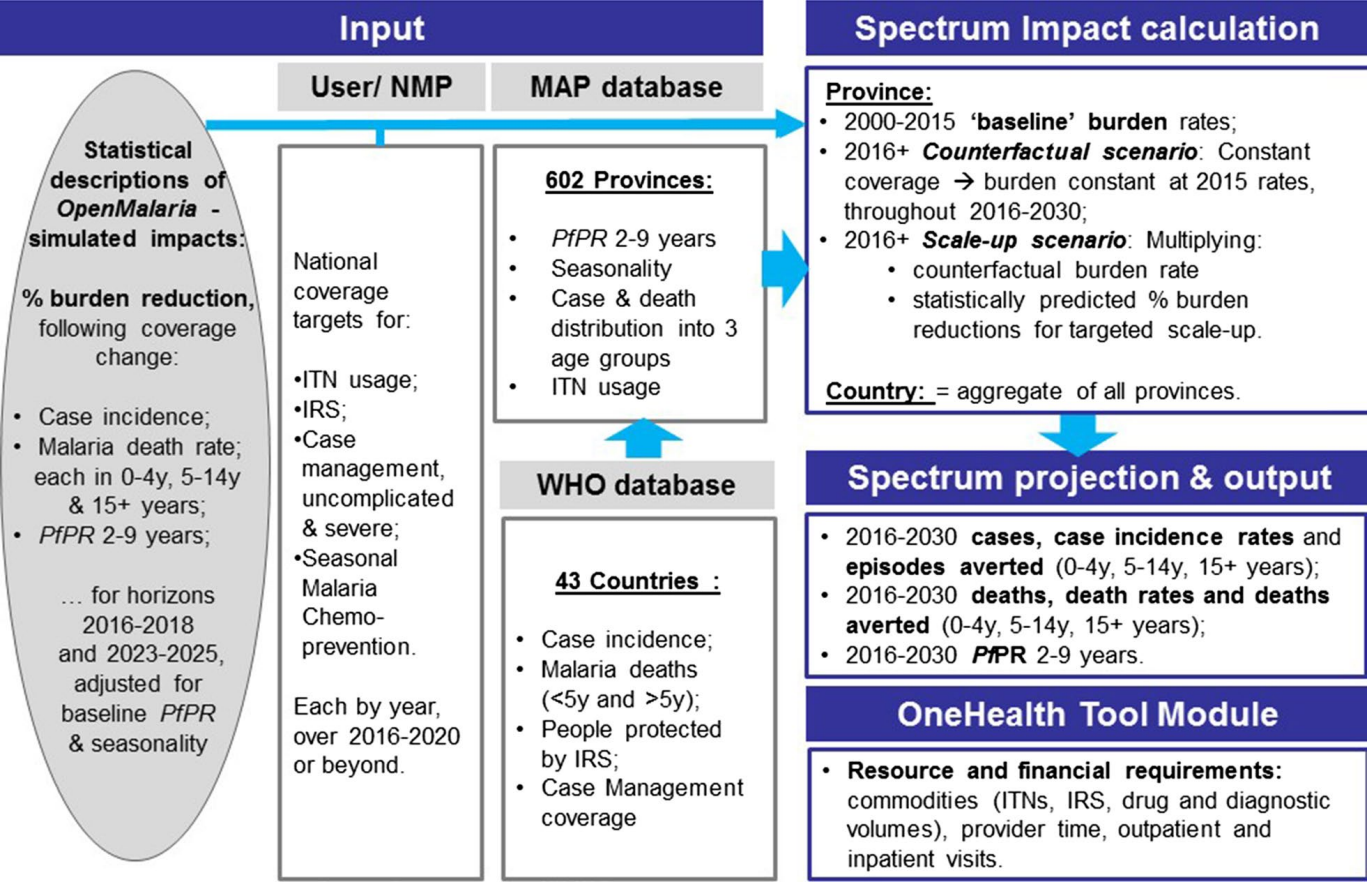
Design of the Spectrum-Malaria impact module

Spectrum applies these functions to first administrative level (Admin1) units (i.e., states or provinces) in a country [17], using Admin1-level endemicity, baseline burden rates and baseline coverage values available from the WHO and malaria atlas project (MAP) [2, 18, 19], in turn for a scenario of user-specified, intervention scale-up and a counterfactual of constant coverage. Proportional burden reductions associated with the user-specified scale-up are then aggregated across Admin1 units, and summarized as population-weighed, national proportional burden reductions, separately for two time horizons of interest (2016–2021 and 2022–2030). Next, burdens are projected over 2016–2030 for country populations defined in Spectrum’s module *DemProj*, by applying the relevant statistically predicted proportional burden reduction (separately for each age group, horizon and health outcome) to expected future counterfactual burdens for a scenario of constant coverage (Fig. 1).

The software package can be downloaded for free [20], with a user manual about installation and application available [21].

### Country data

For 602 Admin1 units of 43 countries in sub-Saharan Africa, Spectrum was pre-populated with annual data over 2000–2015, described below and summarized in Table 1.

**Table 1:** Country and sub-national data pre-loaded in Spectrum-Malaria

Indicator	Age group	Unit used in Spectrum	Data source and comment
*Pf*PR	2–9 years	% of children infected, as weighed median for the Admin1 population, *after excluding the population living at 0.0 PfPR*	*MAP.* The category with 0 *Pf*PR, and its exclusion from the Admin1′s weighted median *Pf*PR, are needed to convert burden and coverage rate between the population with > 0 *Pf*PR in Spectrum (onto which OpenMalaria-based impact regressions are applied) and WHO official country estimates which includes populations with 0 *Pf*PR
Case incidence	0–4, 5–14 and 15+ years	Population-weighted average rate, *for population living at* >*0 PfPR* ^a^	*WHO* national estimates, allocated across Admin1 s, according to case incidence and population size distributions estimated by MAP for that year. 2014 rates applied in 2015
Malaria-attributable mortality	<5 and ≥5 years	Population-weighted average rate, *for population living at* >*0 PfPR* ^a^	*WHO* national estimates for 0–4 vs 5+ years, allocated by Avenir Health to Admin1 s and the 5–14 vs 15+ years sub-age groups, according to distribution in case incidence from MAP for that year. 2014 rates applied in 2015
Seasonality index CV_MAP_EIR	N/A	Population-weighted average, *for population living at* >*0 PfPR* ^a^	*Swiss TPH and MAP* See Additional file 1. Time-constant
Population who slept under an ITN last night	All age total	Population-weighted average, in two variants: 1. Actual (including *population living at 0 PfPR* ^a^ *)* to inform ITN distributions for costing; 2. Effective coverage (limited to *population living at* >*0 PfPR* ^a^ *),* used for impact calculation	*MAP and WHO* [31, 32] N.B. Unlike Bhatt and colleagues’ 2015 national-level estimates, the MAP and Spectrum ITN coverage data at pixel and Admin1 unit level did not assume that ‘*all ITNs are distributed among households situated in malaria*-*endemic regions*’
IRS: % of people protected	All age total	Population-weighted average, in two variants: National (including *population living at 0 PfPR* ^a^, for whom 0 ITN usage is assumed); Effective coverage (limited to *population living at* >*0 PfPR* ^a^ *)*	*WHO.* Allocated from WHO’s national total to Admin1 s through the algorithm shown in Table 3 For 2015, calculate coverage based on the WHO-reported number of people protected in 2014
Case management coverage, uncomplicated cases	All age total	Population-weighted average, constant across all Admin1 units in a country, *irrespective of PfPR*	National estimate for 2015 of the proportion of fevers in children 0-4 years treated with an ACT [26], applied at 2014 and 2015 and to all age groups.
Population size	0–4 years, 5–14 years, 15+ years	Total, *including people at* >*0 PfPR*	*MAP*, for both total population, and population at >0 *Pf*PR

*Pf*PR at province level is calculated on all pixels in the province with *Pf*PR >0. It is the weighted median *Pf*PR over all these pixels, with pixels weighted by their population aged 2–9 years. Avenir Health prepared but did not use an alternative method: the populations of all pixels at *Pf*PR >0 are pooled, with each ‘person’ in the pool assigned their pixel’s *Pf*PR, and taking the median *Pf*PR of the pool. The difference is less than 1% for most provinces, therefore the more intuitive method was selected

Case incidence rates are calculated using whole population (not population at *Pf*PR >0). MAP’s case incidence is always zero for pixels at *Pf*PR = 0; same for Avenir Health’ interpolated malaria death rates, because where there are no malaria cases there can be no malaria deaths

ITN coverage is calculated for both the whole population (‘ITNactual’ in Spectrum’s user interface) and for the population at *Pf*PR >0 (‘ITNeff’)

IRS coverage is being calculated with the whole population as denominator, rather than population at *Pf*PR >0

The number protected by IRS in 2015 is taken to be that reported by NMPs to WHO in 2014 or if 2014 not available, then 2013. If the values in both 2013 and 2014 are missing or zero, Spectrum assumes 0 IRS coverage in 2015 too

Allocation of national IRS numbers protected across provinces is inversely proportion to the past three years’ average of ‘ITNeff’

Seasonality values are calculated across all pixels (i.e., not as ‘effective’ Seasonality on pixels with *Pf*PR >0 only)

^a^Spectrum impact projections are done using as driver/predictor, the *effective coverages in populations with PfPR* >*0,* because only populations with *Pf*PR >0 were simulated in OpenMalaria, and coverage-impact relationships derived from OpenMalaria thus apply to populations with malaria transmission (i.e. *Pf*PR >0, in most years) only

### Case incidence

Country estimates [as opposed to National Malaria Programme (NMP) and health management information system (HMIS) reports of cases and deaths, which in most countries under-state population-level burdens] were obtained from WHO’s Global Malaria Programme, for malaria case incidence (comprising uncomplicated and severe cases) and malaria-attributable deaths [1].

For most sub-Saharan African countries with stable falciparum malaria, WHO’s case and death estimates were based on an epidemiological model that projects case incidence from parasite prevalence measured through household surveys [2]. For 12 mostly lower-endemic countries, WHO estimates are based on NMP-reported clinical cases, with adjustment for (public) clinic coverage and reporting completeness, which sometimes resulted in case incidence trends over time that were at variance with those estimated by MAP (see below), and/or with WHO’s mortality trend estimates (giving fluctuating or eccentric case fatality rates). To avoid inconsistencies within Spectrum’s display of historic (pre-projection) burden trends for these 12 countries, Spectrum took WHO-based 2015 case incidence estimate and derived numbers and rates for 2000–2014 by applying the historic trend in case incidence from MAP, assuming either a same fixed difference in annual case numbers over 2000–2014 as in 2015, or a proportional scaling (Additional file 1).

WHO’s all-age-aggregated national case numbers were allocated across three age groups and Admin1 units, using annual age- and Admin1-specific case incidence estimates from MAP, who combined a spatial–temporal statistical description of *Pf*PR (see below) with a statistical model of the *Pf*PR-case incidence relationship, fitted to simulations from OpenMalaria and two other transmission models and accounting for local history of exposure and treatment coverage [18].

### Malaria-attributable deaths

WHO’s death estimates are available for up to 4 vs 5 years and above. Spectrum applied a 5–14 vs 15+ years breakdown, as for case incidence in MAP’s 2015 estimates [18]. This produced case fatality rates consistent with epidemiological meta-analyses [22], with 90% of values across the 602 Admin1 units ranging from 0.13 to 0.54% for children up to 4 years, and 0.04–0.36% for children 5–14 years and adults.

### *Plasmodium falciparum* infection (*Pf*PR)

*Plasmodium falciparum* infection in children 2–9 years, the core malaria indicator used by MAP, was used as an endemicity/transmission intensity indicator. Admin1-level *Pf*PR in children 2–9 years was derived from MAP’s time-dynamic maps, at 5 × 5 sq km pixel resolution. MAP generated these spatio-temporal ‘cubes’ via statistical modelling, informed by geo-positioned prevalence surveys, intervention coverages and treatment-seeking behaviour, plus a bespoke suite of high-resolution spatial and temporally dynamic predictor variables tracing environmental and sociodemographic determinants [23]. For Spectrum, pixels *within* the limits of stable transmission were aggregated to give Admin1 population-weighted totals and averages using an Admin1 shape file of 2013 [17]. Across the 43 countries, the average number of Admin1 units was 14, ranging from 6 (Djibouti) to 37 (Nigeria).

Statistical impact functions used, as the key predictor variable indicating endemicity, the OpenMalaria-simulated *Pf*PR averaged over 2000–2002, i.e., largely before intervention scale-up started to reduce *Pf*PR [16].

### Seasonality

As an additional endemicity-related impact modifier, Spectrum considers seasonality in malaria transmission, estimated by MAP of a mono-modal yearly pattern based on daily rainfall data over 1980–2010 [24, 25] (Additional file 2). The MAP seasonality index (MAPSI) is a continuous variable. A value of 1 represents an EIR time series that, when log-transformed, is sinusoidal with a semi-amplitude of 2.5, while year-round constant EIR gets a MAPSI of 0. The index is capped at 1; for Africa, pixel values ranged from 0 to just below 1 (Additional file 2).

### Coverage of case management, uncomplicated cases

Available estimates that resemble the definition of effective CMU coverage simulated in OpenMalaria were based on caregiver-reported treatment of fevers in children up to 4 years old with artemisinin-based combination therapy (ACT), as periodically measured in national household surveys in most African countries [26]. National coverage estimates for the year 2015 were taken as Spectrum’s coverage at 2014 and 2015 for all Admin1 units in a country, and were applied to all age groups, because data for older people are generally unavailable.

### Coverage of case management, severe cases

In the absence of reliable country data, this was fixed at 48% at 2014–2015 for all countries and Admin1 units, the proportion of severe episodes receiving inpatient care used previously in various models of falciparum malaria epidemiology in Africa, including OpenMalaria [27–30].

### ITN coverage

Spectrum-Malaria uses as ITN coverage indicator the proportion of people of all ages who slept under an ITN, as estimated by MAP and WHO over space and time in a Bayesian compartmental discrete-time model that triangulated: (a) historical ITN production and delivery from manufacturers, by country and year; (b) national ITN distributions reported by NMPs; and, (c) household ITN ownership and usage reported in nationally representative household surveys. From these data, the compartment model inferred functional representations of ITN retention rates, decay, loss and allocation patterns across and within households, which were subsequently applied to ITN ownership and usage in geographical dis-aggregations provided by the household surveys [31, 32].

### IRS coverage

The proportion of people protected by IRS was calculated from WHO-reported people protected by IRS, based on annual reports by NMPs. The latest available data (from 2014, or 2013 if not reported in 2014) were applied to give the coverage proportion for 2014 and 2015. If a country reported no IRS since 2011 or before, coverage was set at 0 for all years until 2015.

Lacking standardized multi-country data of sub-national IRS allocations, Spectrum allocated national IRS coverages across Admin1 units with >0 *Pf*PR at 2015, each with an IRS probability inversely proportional to its ITN coverage in 2015 (Table 2). For each ‘selected’ Admin1, coverage is set at 90% of the resident population; only the last Admin1 unit that gets IRS allocated has a below 90% IRS coverage, so as to match the national number of people protected. If two Admin1 units have the same ITN coverage, IRS gets allocated to the unit with lowest current IRS coverage; if this is equal between two Admin1 units, IRS is allocated to the unit with highest *Pf*PR at 2015. The resulting minimal overlap between IRS and ITNs reflects recommendations of WHO’s 2015 global technical strategy for malaria [4].

**Table 2:** Allocation of national-level people protected by IRS, to Admin1 units: Nigeria 2015

Admin1/state	Population	PfPR % 2–9 years	ITN coverage %	Probability of IRS	People pro-tected by IRS	IRS coverage %
Abia	3,680,378	37	27	73%		
Adamawa	3,914,499	31	18	82%		
Akwa Ibom	4,859,492	40	33	67%		
Anambra	5,642,911	14	24	76%		
Bauchi	5,816,387	30	19	81%		
Bayelsa	1,938,009	19	35	65%		
Benue	5,545,269	28	31	69%		
Borno	5,409,746	19	17	83%		
Cross River	3,696,053	28	39	61%		
Delta	5,109,252	13	25	75%		
Ebonyi	2,673,883	26	38	62%		
Edo	4,583,326	20	23	77%		
Ekiti	3,056,399	43	30	70%		
Enugu	4,238,001	14	28	72%		
FCT - Abuja	1,569,832	33	27	73%		
Gombe	2,996,780	29	19	81%		
Imo	5,025,285	34	27	73%		
Jigawa	5,648,410	19	26	74%		
Kaduna	7,933,232	46	21	79%		
Kano	12,733,799	23	16	84%	316,255	2.5
Katsina	7,692,509	25	25	75%		
Kebbi	4,196,653	41	30	70%		
Kogi	4,107,085	28	30	70%		
Kwara	3,223,696	42	30	70%		
Lagos	14,316,546	5	22	78%		
Nassarawa	2,332,988	32	26	74%		
Niger	5,207,649	41	26	74%		
Ogun	4,248,558	21	36	64%		
Ondo	4,551,442	35	32	68%		
Osun	4,830,341	40	26	74%		
Oyo	7,588,162	33	31	69%		
Plateau	4,071,155	27	25	75%		
Rivers	6,195,103	19	30	70%		
Sokoto	4,607,886	22	19	81%		
Taraba	2,761,408	33	21	79%		
Yobe	3,067,612	20	21	79%		
Zamfara	4,501,061	32	18	82%		
Nigeria national	*183,570,791*	*27*	*25*		*316,255*	*0.17*

The Spectrum algorithm for sub-national IRS allocation first excludes all Admin1 units with 0 *Pf*PR (if any, not applicable in Nigeria). Among Admin1s with >0 *Pf*PR, IRS gets allocated according the highest complement of ITN coverage (=100% − ITN coverage), at 90% IRS coverage (of the population living at *Pf*PR>) for each successive Admin1 unit, until the total people protected across selected Admin1 units saturates to the national total number of people protected. The last Admin1 unit allocated IRS gets a <90% IRS coverage (for Nigeria at 2015: Kano state, with 2.5% IRS coverage), to exactly meet the national total number

### SMC coverage

As default, SMC coverage was set at 0% for all countries and Admin1s throughout 2015. User-specified, national-level, SMC coverage targets are allocated uniformly across all Admin1s with *Pf*PR >0.

### Severe case incidence

For severe case incidence there are no official WHO or MAP estimates. Therefore, severe case burdens were estimated within Spectrum, based on ratios of severe-to-total case incidence from OpenMalaria simulations that informed Spectrum’s coverage-impact functions, through separate statistical functions that used the same predictor variables as for coverage-impact functions. Severe-to-total case incidence ratios were logit-transformed for regression analysis.

Incidence of severe disease in OpenMalaria depends on parasite density rates when otherwise uncomplicated episodes coincide with co-morbidities (modelled in OpenMalaria as an age-dependent risk), and on maternal immunity that partially protects infants from malaria. Model calibration used routine hospital, community-based surveillance, and demographic data from various African sites [30, 33, 34].

In OpenMalaria simulations and Spectrum’s corresponding statistical functions, severe-to-total case incidence ratios were highest in children up to 4 years old, and lowest in adults. The statistical fit (R^2^) of predicted relative to simulated ratios was 98% for 15+ years, 96% for 5 to 14 years and 80% for up to 4 years, at the 2016–2018 horizon (Additional file 3). Using these statistical functions, Spectrum calculates severe-to-total case incidence ratios for three age groups in each Admin1, as a function of the Admin1’s endemicity and intervention coverages at 2015. Resulting Admin1-specific, age-specific ratios are then applied every year throughout the projection, to derive severe case incidence rates from total case incidence rates.

### Projection model

Spectrum-Malaria interacts with two other modules within Spectrum: ‘DemProj’ and the AIDS incidence model (AIM). Demographic projections are done at country level in DemProj, which interacts with AIM to add HIV/AIDS-related deaths. For the current version of Spectrum-Malaria, mortality reductions caused by malaria control are not fed into DemProj, i.e., malaria mortality or control is assumed to not influence demography.

### National coverage targets, and allocation to Admin1 units

Users must specify targets for effective coverage at national level, up to a maximum of 90% judged to be the maximum feasible coverage. For ITNs, the user-specified (post-)2016 coverage is allocated to Admin1 units, assuming a fixed proportional increase (or decrease) for each Admin1 (Table 2). If any Admin1 gets capped at 90% target coverage, in order to still reproduce the national-level target, the remaining ITNs are re-allocated to another Admin1 with next-highest (but <90%) ITN coverage. IRS coverage targets get allocated to successive Admin1 s with *Pf*PR >0 and lowest concurrent ITN coverage, at 90% each except for the last Admin1 where <90% coverage is fitted to match the national total.

### Impact projection: ITNs, IRS, CMU, and SMC

Spectrum applies statistical impact functions as *proportional* burden reductions, rather than absolute predicted post-scale-up burden levels, because OpenMalaria simulations and the corresponding statistical functions were not calibrated on the WHO and MAP 2015 burden estimates that constitute Spectrum’s baseline data.

In impact functions [16], proportional impacts were generally smaller over the initial (2016–2018) horizon than over an intermediate (2019–2021) and longest (2023–2025) horizons, for scale-up of IRS, ITNs and CMU and their combinations, reflecting that full health impacts are realized from around 3–5 years after reaching high coverage. A translation step was needed in Spectrum to apply these time-varying impacts, based on simulations and predictions for one-off immediate coverage changes (always at 2016), for programme scenarios with multiple interventions scaled-up over different time periods. Requirements were:

1. The algorithm calculates impact over time of multiple interventions scaling-up over successive years, possibly for each intervention in a different time pattern;
2. Projected impacts reflect relevant dynamics over time including partial rebounds due to changing transmission and immunity, as in OpenMalaria and in the statistical functions for three successive 3-year periods following scale-up;
3. For the counterfactual scenario of no change in coverage, burden rates are constant (and absolute numbers of cases and death increase proportionally with population growth);
4. Burden levels in the long-term reflect the final coverages specified, and (as in OpenMalaria and statistical functions) are independent of whether scale-up was at-once, front-loaded, linear, or back-loaded.
5. So as to not overestimate impact in the first and second year of user-specified short-term coverage targets, the impact of any coverage change applies with a 1-year lag (e.g., for ITN scale-up starting at 2016, impact starts in 2017), where the 2016 projection reflects the coverage increase from 2014 to 2015 according to MAP and WHO data.

Spectrum uses statistical impact models of outcomes at one to three years after intervention scale-up throughout the first six projection years (2016–2021), and for 2022–2030 switches to statistical functions for outcomes at 8–10 years after scale-up. This is operationalized as:

$$X\left( t \right) = X\left( {2015} \right)*\frac{{impact\left( {Cov\left( {t_{0} } \right),Cov\left( {t - 1} \right)} \right)}}{{impact\left( {Cov\left( {t_{0} } \right),Cov\left( {t_{0} } \right)} \right)}}$$

where $X$ denotes the outcome projected (cases, deaths, or *Pf*PR), and $impact( \ldots , \ldots )$ returns a statistically predicted burden, as a function of changes in coverages over a specified period between an initial and a target year. The ratio of two statistically predicted burdens, for the scenario of user-specified scale-up relative to the scenario of constant coverages, gives an impact ratio that Spectrum then applies, by updating $X\left( t \right)$ relative to $X\left( {2015} \right)$, starting from $t_{0} = 2014$ for the impact in 2016.

After the combined impact of IRS, ITN, CMU and SMC coverage trends has thus been projected, the trend in malaria mortality is adjusted to reflect the additional impact of CMS coverage, as:

$$Deaths_{Adj} \left( t \right) = Deaths\left( t \right)*\frac{1 - SevereCM(t)}{1 - SevereCM(2015)}$$

which is applied for each Admin1 unit, using Admin1-specific endemicity and baseline coverages, for all $t$ from 2016 through the end of the projection. Finally, national-level outcomes for each age group are produced by aggregation across Admin1 units.

### Impact projection: malaria mortality

Spectrum-Malaria aligns with the one-death-one-cause framework of LiST and the mortality estimates of the United Nations Child Health Epidemiology Reference Group (CHERG), WHO and global burden of disease project [1]. Malaria mortality represents deaths directly attributable to malaria, without additional indirect malaria-related deaths to which malaria contributes when concurrent with or preceding another disease. However, especially in young children, indirect malaria-related mortality adds a considerable burden, estimated to double overall malaria-related mortality [34], as illustrated by cluster-randomized ITN trials in endemic Kenya and Ghana, where ITN scale-up reduced all-cause under-five mortality almost as much as malaria-attributed mortality [35–38]. In order to not understate the mortality impacts possible through malaria control, yet respect the one-death-one-cause framework, the lives-saved models used by CHERG, LiST, WHO, and the global burden of disease estimated a proportional malaria-related mortality reduction that they apply to direct malaria-attributable deaths (e.g., in LiST: a 55% reduction, at 90% ITN ownership or 60% child ITN usage [12, 13]), which corresponds to the observed 17% reduction in all-cause under-five mortality reductions observed in the ITN trials [38]. While the OpenMalaria simulations that informed Spectrum’s impact functions simulated direct malaria-attributable and indirect malaria-related mortality separately, statistically predicted reductions in direct malaria-attributable deaths in up to 4 years old children were in line with CHERG-estimated reductions for malaria-related mortality used in LiST [16]. Therefore, Spectrum uses the OpenMalaria-based proportional reductions for direct malaria-attributable deaths.

### Impact projection: severe case management

For CMS, Spectrum projects an impact on malaria mortality rates in the three age groups, without dynamic impacts on any of the other burden indicators, or on mortality in years beyond that of coverage shift concerned. Up to 2015, coverage of CMS is assumed to be a fixed 48% across age groups and Admin1 units, as in OpenMalaria [30]. The effect of increasing CMS coverage to a user-specified target is calculated based on an assumed threefold relative risk of mortality between severe cases effectively treated and severe cases not effectively treated, based on corresponding odds ratios of 2.1 assumed in OpenMalaria [30] and 5 in LiST (considering that intravenous quinine reduces malaria mortality in children 1–59 months by 82% compared to no treatment [39]). Spectrum’s threefold relative risk, with a baseline CMS coverage of 48%, translated into a maximum 41% reduction in malaria mortality when CMS is scaled-up to the maximum coverage of 90%. Corresponding proportional mortality reductions are interpolated linearly for more moderate scale-up.

This calculation is repeated for each age group and year, at country-level since CMS coverage and effectiveness assumptions do not differ among Admin1s within a country), starting from the severe case incidence rate and malaria mortality rate for that age group and year as projected based on target coverages of ITN, IRS, CMU, and SMC.

In contrast to the four other interventions for which impacts include onward dynamic long-term transmission effects (based on OpenMalaria dynamic simulations), projected impact is immediate for CMS within the year of coverage change.

## Results

**Fig. 2:**
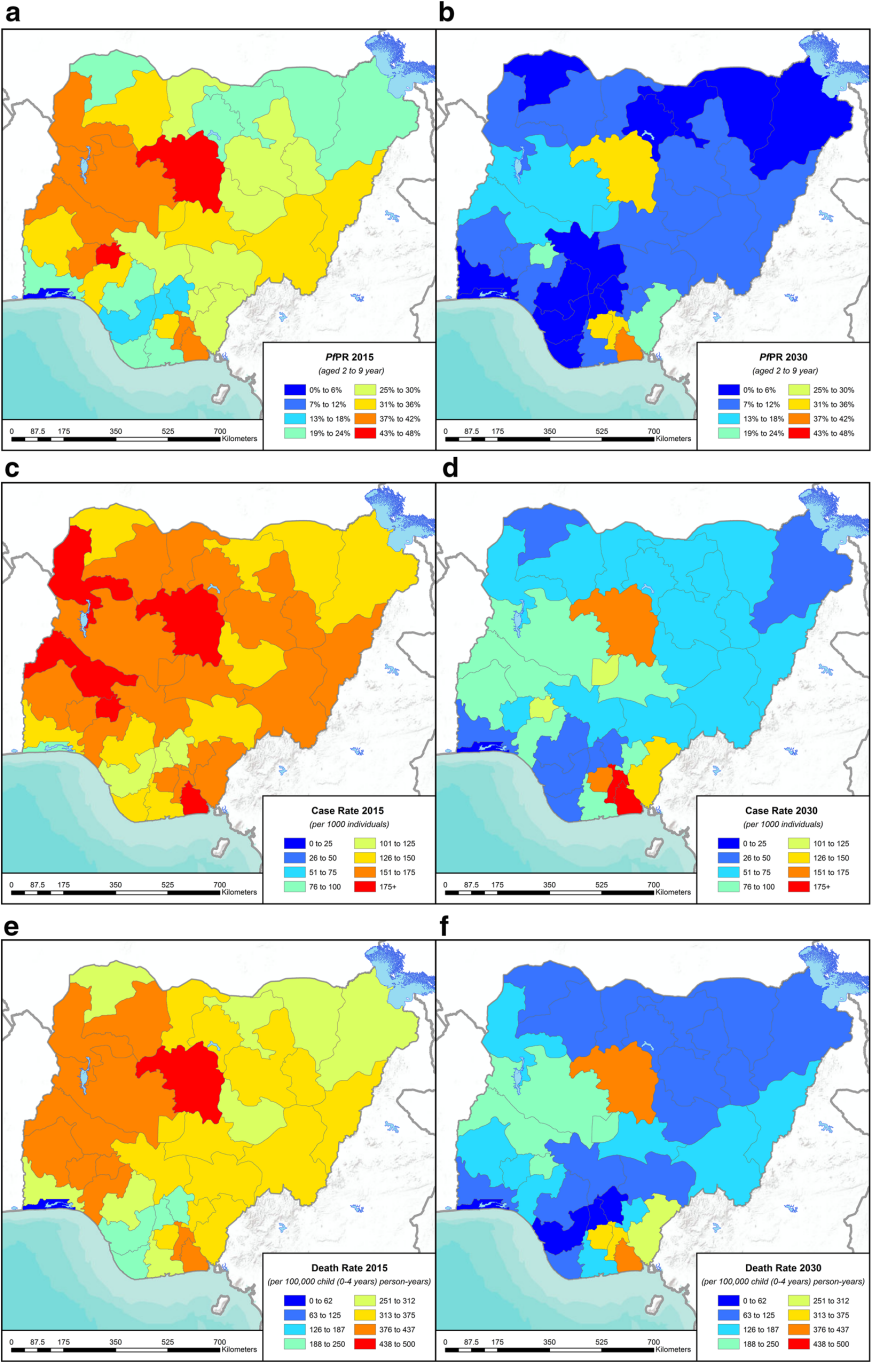
Malaria health burdens at 2015 baseline and at 2030 after scale-up of ITN coverage to 80% usage from 2020 onwards, in Admin1 units of Nigeria. **a**, **b** *Pf*PR in children 2–9 years; **c**, **d** malaria case incidence rate in adults 15+ years; **e**, **f** malaria deaths in children 0–4 years

Projections were performed for Nigeria, using the default data pre-loaded in Spectrum (Table 1; Fig. 2). Table 2 illustrates Spectrum’s allocation of WHO-reported national IRS coverage into Admin1-level coverages at 2015 in Nigeria, provided as default, for users to subsequently refine with locally informed updates.

**Fig. 3:**
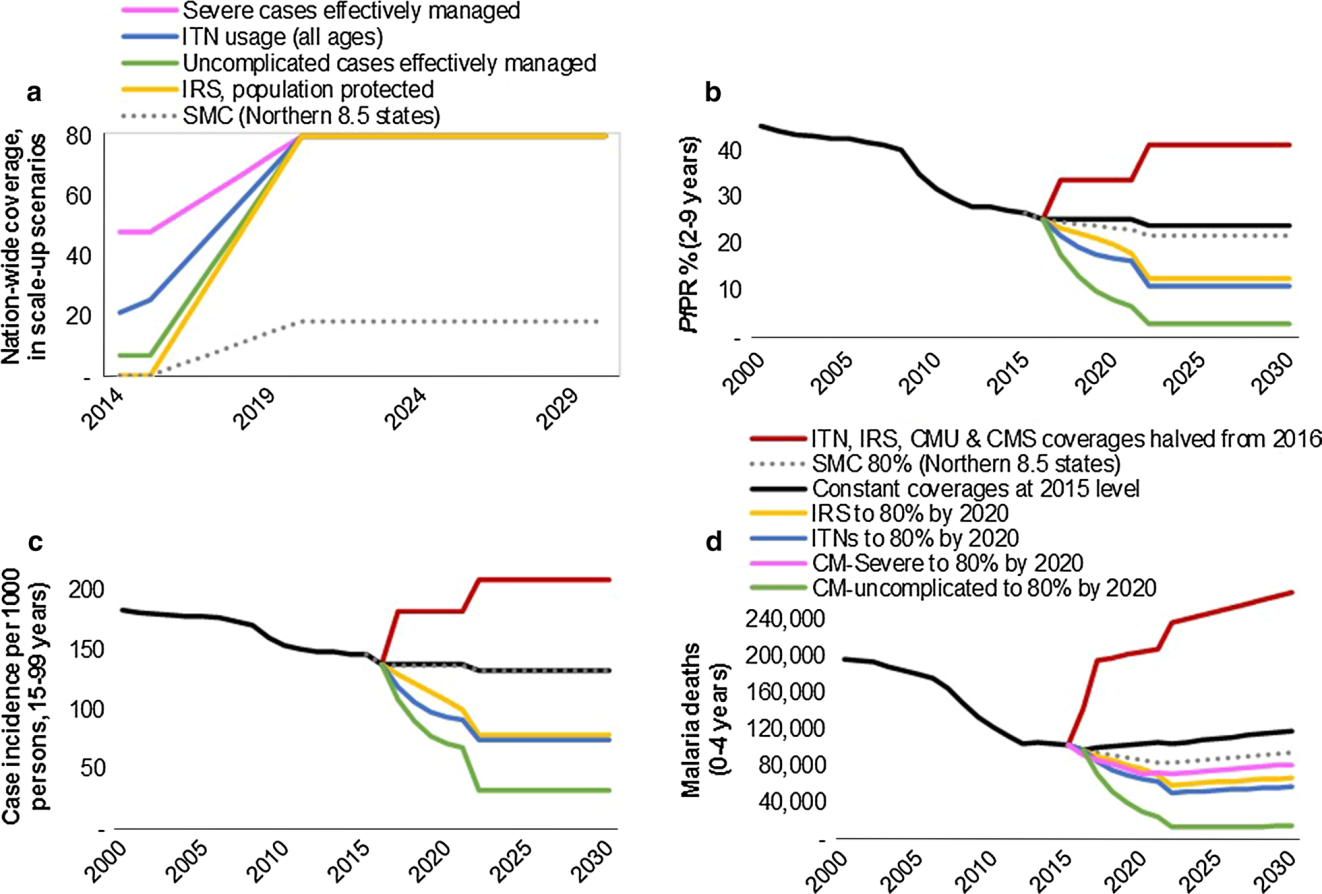
Spectrum-projected impacts of malaria intervention scale-up in Nigeria, on selected health outcomes. **a** Coverage scale-up; **b** *Pf*PR in children 2–9 years; **c** malaria case incidence rate in adults 15+ years; **d** malaria deaths in children 0–4 years. Coverage definitions as specified in “Methods” and Table 1. For SMC, coverage was scaled-up to 80% in eight northernmost states and to 40% in Bauchi state, yielding a nationwide coverage of about 18% from 2020

Impacts were projected for scale-up of ITNs, IRS, CMU, CMS in turn, each to 80% national coverage by 2020, in linear scale-up from their 2015 baseline levels (Fig. 3a), relative to a projection with constant coverages for all interventions. A fifth scenario projected SMC scaled-up to 80% by 2020 in the states Kebbi, Sokoto, Zamfara, Katsina, Kano, Jigawa, Yobe, and Borno, and to 40% in Bauchi (to represent this state’s northern half), in line with WHO recommendations on the geographical applicability of the intervention [40]. A final projection halved the coverage of ITNs, IRS, CMU, and CMS, all four at once from 2016.

ITNs, IRS and CMU each reduced *Pf*PR and case incidence and malaria mortality in all age groups, with the largest impacts (for scaling to 80% coverage) for CMU, followed by ITNs and IRS (Fig. 3b–d). CMS (scaled from 48 to 80%) reduced malaria mortality (but not *Pf*PR or case incidence) by a smaller extent, but starting a year earlier. SMC (reaching 18% coverage nation-wide, from scale-up in 8.5 states) had negligible impact on *Pf*PR and case incidence in adults, but reduced under-five deaths.

Although the coverage increase assumed for Nigeria was larger for IRS (from 0.17 to 80%) than for ITNs (from 25 to 80%), projected impacts were slightly larger for ITNs. This is in part because the incremental impact of IRS is constrained by the pre-existing moderately high (25%) ITN coverage, as the statistical impact functions include a saturation effect [16], whereby the scale-up of IRS and ITN combined in an Admin1 result in a relatively smaller incremental impact than the sum of individual intervention scale-ups, in line with the latest insights and recommendations for vector control by the WHO [41].

Proportional case and death reductions were largest for children up to 4 years (Table 3) but also considerable for children 5–14 years and adults 15 years and above for each intervention (Table 3); only SMC impact was limited to cases and deaths in up to 4 years and *Pf*PR 2–9 years. Corresponding reductions in absolute numbers of infections and deaths averted were much larger in children under 5 years old, reflecting their higher baseline burden.

**Table 3:** Health outcomes projected for Nigeria, during scale-up of ITN coverage from 27% at 2015 to 80% from 2020

Year	2014	2015	2016	2017	2018	2019	2020	2021	2022–2030^a^	Unit	Rate ratio, 2022/2015	Absolute numbers, 2015
Age 0–4 years												
All cases	873	866	812	691	607	551	512	486	341	Per 1000	0.39	27,851,814
Uncomplicated cases	800	794	745	635	558	507	472	448	316	Per 1000	0.40	25,532,547
Severe cases	73	72	67	56	49	44	40	38	24	Per 1000	0.33	2,319,267
Deaths	335	318	299	255	224	203	187	176	141	Per 100,000	0.44	102,368
Age 5–15 years												
All cases	380	374	351	301	266	245	231	223	185	Per 1000	0.50	18,439,829
Uncomplicated cases	370	364	342	293	260	239	226	218	182	Per 1000	0.50	17,959,175
Severe cases	10	10	9.1	7.6	6.5	5.8	5.4	5.1	3.4	Per 1000	0.35	480,654
Deaths	15	15	14	12	11	10	10	10	10	Per 100,000	0.66	7484
Age 15+ years												
All cases	146	146	137	118	105	98	93	91	75	Per 1000	0.51	14,890,633
Uncomplicated cases	145	145	136	117	105	97	93	91	74	Per 1000	0.51	14,800,267
Severe cases	0.9	0.9	0.8	0.7	0.6	0.5	0.4	0.4	0.3	Per 1000	0.33	90,365
Deaths	5.9	5.9	5.5	4.6	4.1	3.7	3.5	3.4	3.9	Per 100,000	0.66	6043
All ages												
All cases	336	333	312	266	234	214	200	192	148	Per 1000	0.44	61,182,276
Uncomplicated cases	321	318	297	254	224	205	192	184	143	Per 1000	0.45	58,291,990
Severe cases	16	16	15	12	10	9.3	8.5	7.9	5.0	Per 1000	0.32	2890,286
Deaths	66	63	59	50	43	39	36	34	28	Per 100,000	0.44	115,894
PfPR 2–9 years	27	27	25	22	19	18	17	16	11	%	0.41	

^a^Same projected annual rates every year over 2022 throughout 2030

For all interventions and health outcomes projected for Nigeria, impacts were enhanced at the 2022–2030 horizon relative to the 2016–2021 horizon, reflecting the typical temporal dynamics in intermediate-endemicity settings, such as Nigeria. Halving the coverage of all interventions resulted in a near doubling of case incidence and *Pf*PR, and a more than doubling of malaria mortality, also with effects most pronounced in the longer term.

Projected reductions in severe case incidence were larger than that in overall case incidence (a lower rate ratio, second-last column of Table 3), despite Spectrum’s application of time-constant ratios of severe-to-total cases. This shift reflects that interventions reduced case incidence proportionally more so in Admin1 units with high severe case rates, i.e., Admin1 units with lower endemicity.

In the long term, intervention scale-up reduced mortality rates slightly less than case incidence rates, increasing the case fatality, from around 0.37–0.41% in children up to 4 years, and from 0.04 to 0.05% in children and adults 5 years and older (Table 3).

## Discussion

Spectrum-Malaria presents a new evidence- and consensus-based approach to predict morbidity and mortality impact of malaria intervention scale-up in adults and children, and to inform strategic programme planning in African countries. The tool’s key input and output measures align with monitoring indicators commonly used in programme and grant performance frameworks; its graphical, menu-driven interface embedded in Spectrum’s demographic platform facilitates relatively quick comparison of programme scenarios, to stimulate local capacity building in surveillance and policy evaluation and stakeholder dialogue. The anchoring on WHO and MAP baseline data ensures relevance for progress evaluation for national plans, global targets such as the sustainable development goals (SDGs), and donor grant performance frameworks, as well as comparability across countries. These features come at the price of user flexibility: users cannot modify baseline burdens and coverages, or the expected future burden trends for counterfactual, constant-coverage scenarios. If users wanted to change country baseline data, the process would be via a country government’s official update for the next year’s World Malaria Report coordinated by the WHO, being reflected in Spectrum-Malaria the year after.

In projections for Nigeria (as well as for other large and highly malaria-endemic countries, presented elsewhere [42]), malaria prevention and treatment reduce health burdens in all age groups, with slightly larger proportional impacts in children under-five, and the largest absolute number of cases and deaths averted in this vulnerable group. For Nigeria, given high ITN coverage but low effective case management coverage in 2015, case management is the intervention for which scale-up to near-universal coverage could have most additional impact by 2030. However, it is often easier to achieve high-level coverage for vector control interventions (often delivered through vertical programmes, as campaigns) than for effective CMU (through complex multi-layer health systems), so this ranking does not imply that CMU is necessarily a more cost-effective investment than vector control. In fact, over 2000–2015, scale-up of ITNs, which reached higher coverage levels than CMU, has been estimated to have had the larger actual impact to date [2]. Programmatic inputs and resources to achieve further scale-up of ITNs, IRS and/or CMU, and the resulting cost-benefit relationships can be assessed using the OneHealth Tool which is linked to Spectrum, as in investment case analyses for other health areas [9, 43], thus enabling evaluation of impacts and costs of malaria interventions alongside, with their trade-offs in short and longer term.

### Strengths and weaknesses

Spectrum-Malaria presents important refinements compared to existing malaria programme planning and impact evaluation tools, including dynamics over time, morbidity outcomes, outcomes in adults, and saturation and synergies across interventions through endemicity effects. As with any model, results are only as valid as the input data and assumptions. Notable limitations in data include baseline rates of severe case incidence, and CMU and CMS coverage, with scale-up targets for CMU and CMS also difficult to define. Estimates of CMU coverage could be improved considering variations sub-nationally and possibly by age, and considering patient compliance, timeliness and dosage [26, 44, 45].

Spectrum-Malaria extrapolates the age pattern in malaria mortality between 5 and 14 years and 15 years and older from MAP-estimated age patterns in case incidence, which implicitly assumes that coverage and effectiveness of CMS and CMU are constant throughout the above-5 years population. If in reality CMU and/or CMS had better coverage in adults than in school-age children, then Spectrum may overestimate mortality, and underestimate future possible treatment impacts on mortality, in adults compared to school-age children. A new refined map of CMU coverage in Africa over 2000–2015, used to generate corresponding age-specific malaria mortality estimates at 5 × 5 sq km resolution, recently produced by MAP [46] after the current design of Spectrum, will be integrated into the baseline database of Spectrum-Malaria at its next annual data update.

Spectrum’s effectiveness estimates for ITN and IRS impact were calibrated on results of three ITN trials; for child mortality, these align with international consensus and estimates used by CHERG, WHO, the Global Fund to fight AIDS, Tuberculosis and Malaria, and the global burden of disease [12, 13, 38, 47, 48]. However, the spread of insecticide resistance threatens to reduce effectiveness, even if resistance indicators available routinely from National Malaria Programmes do not, as yet, allow a meaningful resistance adjustment in Spectrum-Malaria [49]. For CMU and CMS, effectiveness estimates are even less certain, lacking randomized trials in the first place.

A structural limitation in the tool is the assumed time pattern in impact following intervention scale-up, drawn in a simple way from results of schematized, one-time coverage increases and decreases simulated in OpenMalaria. The assumed 1-year lag from coverage increase to start of impact may be conservative compared to other planning tools that project impacts to start immediately, however, for strategic planning purposes and evaluations over a multi-year horizon this should result, if anything, in more robust and realistic longer term impacts since statistically predicted reductions were averaged in 3-year intervals following scale-up. The use of statistical functions for outcomes at 1–3 years and 8–10 years following OpenMalaria-simulated scale-up is also conservative, because OpenMalaria-simulated burden reductions were largest at 4–6 years following scale-up [16]. Nevertheless, Spectrum-projected impacts were well-aligned with those for under-five mortality by LiST [42], which was in turn validated against impacts observed in vector control studies in four African countries [50], and with empirically based ecological analyses of historic impacts on *Pf*PR and case incidence due to ITN, IRS and CMU scale-up over 2000–2015 [2], with if anything, slightly larger long-term impacts reflecting the dynamics of reduced transmission.

Spectrum’s switch from short-term (1–3 years) to long-term (8–10 years) impact functions at 2022 for all policy scenarios, regardless of the setting-specific time pattern of scale-up, should produce valid impacts over time for typical scenarios where interventions are scaled-up gradually from 2016 until a final coverage target achieved no earlier than 2019. However, for scenarios with much earlier or later scale-up, impacts might be more reliably predicted by dynamic simulations for the specific user-specified time pattern of scale-up (using OpenMalaria or other transmission dynamic models [51]), and the validity of Spectrum-Malaria remains unconfirmed.

For counterfactual projections, Spectrum by default assumes that if coverage is unchanged, so is burden, in contrast to empirical analyses suggesting a secular decline over 2000–2015, for a hypothetical zero ITN, IRS and CMU coverage scenario used in MAP’s recent impact evaluation for the millennium development goals [2]. If this trend, thought to reflect changes in urbanization, housing quality, land-use, nutrition, and other socio-demographical factors, is real and continuing after 2015, Spectrum may overestimate future malaria burdens preventable by malaria control, although it would remain accurate for proportional burden reductions.

Finally, while projected impacts include a reduction in *Pf*PR among children aged 2–9 years, Spectrum assumes no change after 2015 in proportions of the population exposed and not exposed to malaria transmission. This simplification could cause Spectrum to underestimate health impacts and overestimate commodities and resources needed for malaria control, especially in the longer term, if successful control reduces the areas of ongoing malaria transmission.

Collectively, these limitations and uncertainties point to the need for ongoing further validation of the tool. While impact predictions were adequately calibrated on observations from ITN trials, and on a range of predictions in the underlying OpenMalaria model (which in turn was previously calibrated by comparison and alignment with extensive programme field data on age and exposure patterns of malaria infection prevalence and disease, including effects of intervention scale-up, in sub-Saharan African settings with stable endemic malaria [15, 52]), it is essential to further compare and where necessary adjust Spectrum-Malaria’s impact projections against longitudinal, multi-year data from large (province or national-level) programmes that achieved high coverage of one or more interventions, and have a good record of baseline (pre-intervention) health burden levels and/or health burden measurements in a comparison (no-intervention) area. The team is planning to undertake such validations as opportunities will allow. Meanwhile model applications to support NMPs in strategic planning, target setting and prediction of expected future health impacts will be carried out with due caution to inevitable uncertainties in results. A next version of Spectrum-Malaria will, therefore, imperatively include uncertainty ranges on projection outcomes, generated by combining error margins in WHO- and MAP-estimated burden and coverage numbers with confidence bounds on statistical impact predictions, to underscore the uncertainty (as with any model) in numerical outcomes.

## Conclusion

The above-stated limitations of Spectrum-Malaria suggest several desired refinements, and more to be expected based on users’ feedback, as the tool gets applied across divergent settings in terms of epidemiology and policy debate. For Nigeria, projections are expected to change once local experts validate and update the assumptions and input data. After on-site validation with Nigeria and the Democratic Republic of the Congo, where a pilot was completed, Spectrum-Malaria could potentially be applied to evaluate the feasibility of, and progress toward the ambitious global targets set by the WHO, Roll Back Malaria and SDGs to reduce case incidence and mortality by 80% by 2025 and by 90% by 2030 [4].

To allow cost-effectiveness and investment case analysis, Spectrum-Malaria links to the OneHealth Tool that projects resource and commodity needs for user-specified programme and impact scenarios [9]. Articulating the cascade of service inputs and activities required to achieve certain effective coverage levels in a OneHealth Tool-supported malaria costing should help users to set realistic rather than over-ambitious targets. For example, effective CMU requires health care access, diagnosis and treatment; effective ITN usage requires ITN distribution as well as community-based education.

The current Spectrum-Malaria is applicable only for countries with stable endemic falciparum malaria, i.e., most of sub-Saharan Africa (and Haiti, if country baseline indicator data became available in MAP-like format). Within sub-Saharan Africa the full range of low to high endemicities was modelled, down to EIRs as low as one infectious bite per adult per year and *Pf*PR 0.1%; intervention scenarios simulated include several nearly achieving elimination. Low-endemicity sites and near-elimination scenarios will become increasingly relevant for strategic planners over next years, as programmes successfully reduce malaria transmission. As malaria control advances, a Spectrum version for settings with predominantly non-falciparum and epidemic malaria will be envisioned, which could build on an OpenMalaria variant for *P. vivax* malaria being developed.

## Additional files

**Additional file 1.** Adjustment of 2000-2014 malaria case numbers from WHO estimates to match the MAP-estimated time trend, for 12 countries.

**Additional file 2.** Conversion between the seasonality index mapped for Africa by MAP, and seasonality index used by OpenMalaria simulations and statistical impact predictions in Spectrum.

**Additional file 3.** Multivariate regression models predicting the ratio of Severe-to-Total incident malaria cases: coefficients and p-values.

## Abbreviations

ACT: artemisinin-based combination therapy
CHERG: United Nations Child Health Epidemiology Reference Group
CMU: effective management of uncomplicated malaria cases
CMS: effective management of severe malaria cases
EIR: entomological inoculation rate
HMIS: health management information system
IRS: indoor residual spraying
ITN: insecticide-treated mosquito net
LiST: lives saved tool (=child survival/mortality impact model)
MAP: malaria atlas project
MAPSI: malaria atlas project’s seasonality index
NMP: National Malaria Programme
*Pf*PR: prevalence of *Plasmodium falciparum* parasite infection
SMC: seasonal malaria chemoprevention
SDGs: sustainable development goals
Swiss TPH: Swiss Tropical and Public Health Institute
WHO: World Health Organization
