# Supplementary material for: Spectrum-Malaria: a user-friendly projection tool for health impact assessment and strategic planning by malaria control programmes in sub-Saharan Africa
Source: Malar J. 2017 Feb 10;16:68. doi: 10.1186/s12936-017-1705-3 (PMC5301449; doi:10.1186/s12936-017-1705-3)

**Additional file 1.** **Adjustment of 2000-2014 malaria case numbers from WHO estimates to match the MAP-estimated time trend, for 12 countries**

For 12 mostly lower-endemic countries, WHO case estimates were based on NMP-reported clinical cases, with adjustment for (public) clinic coverage and reporting completeness, which sometimes resulted in case incidence trends over time that were at variance with those estimated by MAP, or with the WHO’s mortality trend estimate (giving fluctuating or eccentric case fatality rates). To avoid inconsistencies within Spectrum’s baseline specifications, for Eritrea, Ethiopia, Gambia, Mauritania, Rwanda and Senegal, Spectrum took WHO’s 2015 case incidence estimate, and derived numbers and rates for 2000–2014 by applying the historic trend in case incidence from MAP, assuming the same fixed difference in annual case numbers over 2000−2014 as for 2015 (purple lines with dots, in Supplementary Figure 1).

For Botswana, Madagascar, Namibia, Swaziland, South Africa, Zimbabwe, the best fit to MAP’s 2000-2015 time trend was obtained by scaling the WHO 2000-2014 case numbers each year proportionally according to MAP’s 2000-2015 time trend, anchored on the WHO’s 2015 case number (yellow lines with dots, in Supplementary Figure 1).

Of note, the WHO and MAP estimates for these countries each come with considerable uncertainty around the point estimates [[1](#_ENREF_1), [2](#_ENREF_2)], and within these uncertainty ranges the WHO, MAP and resulting Spectrum point estimates are not substantively different.

**References for Supplementary Data file 1:**

1. World Health Organization: **World Malaria Report 2015**. Geneva; 2015.

2. Bhatt S, Weiss DJ, Cameron E, Bisanzio D, Mappin B, Dalrymple U, Battle KE, Moyes CL, Henry A, Eckhoff PA *et al*: **The effect of malaria control on *Plasmodium falciparum* in Africa between 2000 and 2015**. *Nature* 2015, **526**(7572):207-211.

**Supplementary Figure 1.**


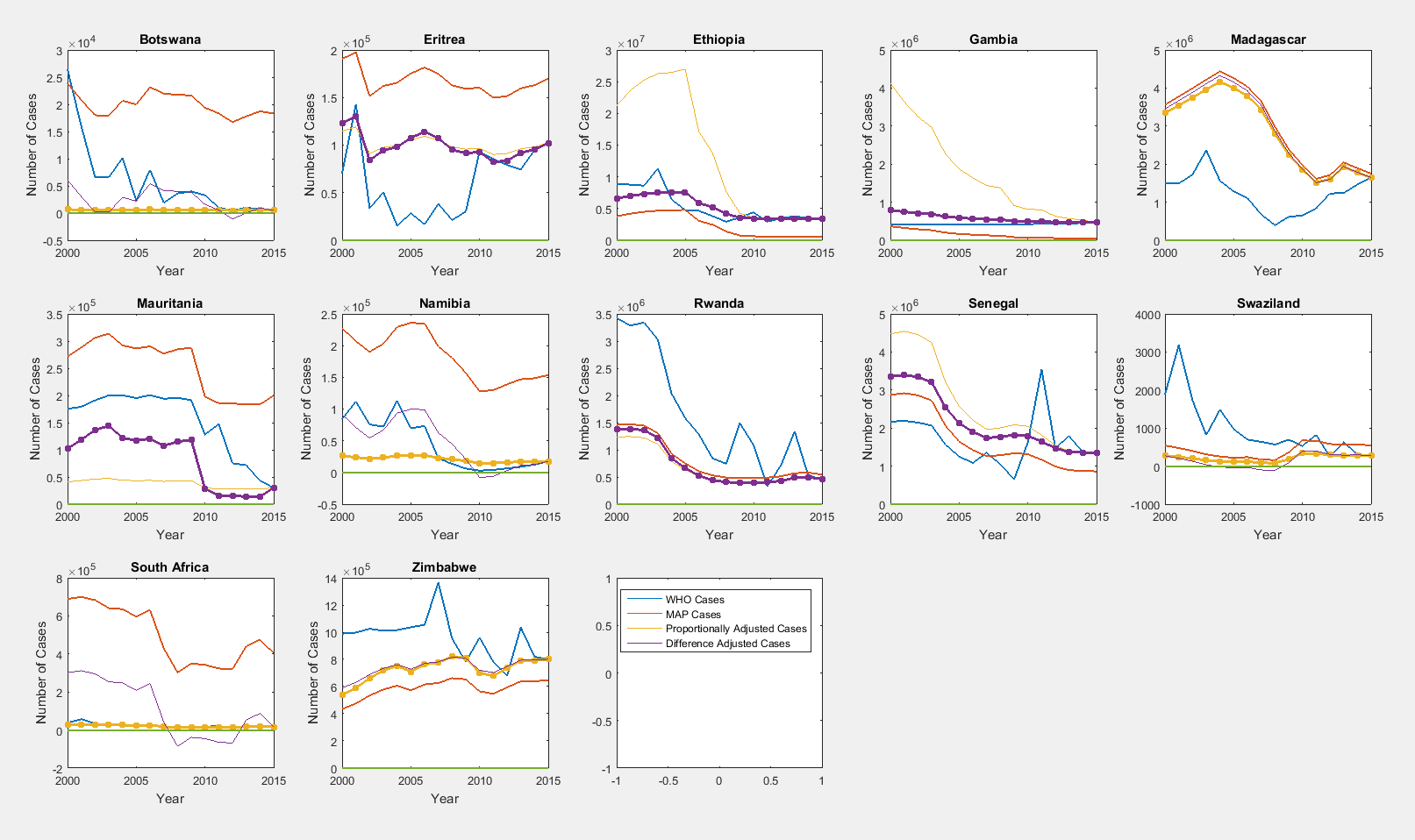

Supplement: Supplementary file 1 — Additional file 1. Adjustment of 2000-2014 malaria case numbers from WHO estimates to match the MAP-estimated time trend, for 12 countries. [file 12936_2017_1705_MOESM1_ESM.docx]
