# Supplementary material for: Spectrum-Malaria: a user-friendly projection tool for health impact assessment and strategic planning by malaria control programmes in sub-Saharan Africa
Source: Malar J. 2017 Feb 10;16:68. doi: 10.1186/s12936-017-1705-3 (PMC5301449; doi:10.1186/s12936-017-1705-3)
Supplement: Supplementary file 2 — Additional file 2. Conversion between the seasonality index mapped for Africa by MAP, and seasonality index used by OpenMalaria simulations and statistical impact predictions in Spectrum. [file 12936_2017_1705_MOESM2_ESM.docx]

**Additional file 2. Conversion between the seasonality index mapped for Africa by MAP, and seasonality index used by OpenMalaria simulations and statistical impact predictions in Spectrum.**

MAP has produced a map for Africa depicting the extent of seasonality according to a mono-modal yearly pattern, based on daily rainfall over 1980-2010 as done previously by MARA [[1](#_ENREF_1)] and analogous to the characterization of seasonality in OpenMalaria [[2](#_ENREF_2)]. The MAP seasonality index (MAPSI) is continuous (floating point). A value of 1 corresponds to an EIR time series that is, when log-transformed, a sinusoidal with a semi-amplitude of 2.5, indicating high seasonality such as in northern Burkina Faso. A flat EIR series (no seasonality) have a MAPSI of 0, indicating no or very little seasonality such as in Equatorial Guinea. The index is capped at 1 (values higher than 1 could occur in "nature", but are set to 1); for Africa, resulting pixels range from 0 to just below 1.

For impact projections in Spectrum, the MAPSI for a given Admin1 area was transformed into the index CV_MAP_EIR used in OpenMalaria simulations and statistical impact regressions [[3](#_ENREF_3)] with the formula:

CV_MAP_EIR = 2.3876 * (1.879747*MAPSI - 0.649789*MAPSI^2).

The resulting CV_MAP_EIR was used to interpolate between simulation and regression outcomes for the OpenMalaria-simulated scenarios with CV_MAP_EIR= 0.1207213 (5% quantile of non-population weighted CV_MAP_EIR in malaria-endemic Africa; OpenMalaria parameter ‘a1’, describing the amplitude of the annual sinusoidal, of 0.171) and CV_MAP_EIR= 2.6614240 (95% quantile; OpenMalaria parameter ‘a1’ of 21.09).

**Supplementary Table 1. Seasonality indices used in OpenMalaria simulations, and values as used in statistical impact functions**

| **Seasonality** | **OpenMalaria parameter ‘a1’ and values used in simulations informing the statistical functions** | **CV_MAP_EIR, in Spectrum** | **MAPSI, from MAP** |
| --- | --- | --- | --- |
| Low seasonal (5% of CV_MAP_EIR in Africa, according to MAPSI for endemic areas) | 0.171 | 0.121 | 0.0272 |
| Moderately seasonal (median of CV_MAP_EIR in endemic areas) | 2.813 | 1.310 | 0.329 |
| Highly seasonal (95% of CV_MAP_EIR in endemic areas | 21.09 | 2.662 | 0.833 |

**References for Supplementary Data file 2:**

1. Mabaso ML, Craig M, Ross A, Smith T: **Environmental predictors of the seasonality of malaria transmission in Africa: the challenge**. *Am J Trop Med Hyg* 2007, **76**(1):33-38.

2. Cairns M, Roca-Feltrer A, Garske T, Wilson AL, Diallo D, Milligan PJ, Ghani AC, Greenwood BM: **Estimating the potential public health impact of seasonal malaria chemoprevention in African children**. *Nat Commun* 2012, **3**:881.

3. Korenromp EL, Mahiané G, Hamilton M, Pretorius C, Cibulskis R, Lauer J, Smith TA, Briët OJT: **Malaria intervention scale-up in Africa: statistical effectiveness predictions for health program planning tools, based on dynamic transmission modelling**. *Malar J* 2016, **15**(417).
