# Supplementary material for: Spectrum-Malaria: a user-friendly projection tool for health impact assessment and strategic planning by malaria control programmes in sub-Saharan Africa
Source: Malar J. 2017 Feb 10;16:68. doi: 10.1186/s12936-017-1705-3 (PMC5301449; doi:10.1186/s12936-017-1705-3)
Supplement: Supplementary file 3 — Additional file 3. Multivariate regression models predicting the ratio of Severe-to-Total incident malaria cases: coefficients and p-values. [file 12936_2017_1705_MOESM3_ESM.docx]

**Additional file 3 : Multivariate regression models predicting the Ratio of Severe-to-Total incident Malaria cases: coefficients and p-values**
